# A Crucial Role of the Frontal Operculum in Task-Set Dependent Visuomotor Performance Monitoring

**DOI:** 10.1523/ENEURO.0524-21.2021

**Published:** 2022-03-02

**Authors:** Felix Quirmbach, Jakub Limanowski

**Affiliations:** 1 Faculty of Psychology, Technische Universität Dresden, 01069 Dresden, Germany; 2 Centre for Tactile Internet with Human-in-the-Loop, Technische Universität Dresden, 01187 Dresden, Germany

**Keywords:** action, frontal operculum, performance monitoring, sensorimotor integration, visuoproprioceptive integration

## Abstract

For adaptive goal-directed action, the brain needs to monitor action performance and detect errors. The corresponding information may be conveyed via different sensory modalities; for instance, visual and proprioceptive body position cues may inform about current manual action performance. Thereby, contextual factors such as the current task set may also determine the relative importance of each sensory modality for action guidance. Here, we analyzed human behavioral, functional magnetic resonance imaging (fMRI), and magnetoencephalography (MEG) data from two virtual reality-based hand–target phase-matching studies to identify the neuronal correlates of performance monitoring and error processing under instructed visual or proprioceptive task sets. Our main result was a general, modality-independent response of the bilateral frontal operculum (FO) to poor phase-matching accuracy, as evident from increased BOLD signal and increased source-localized gamma power. Furthermore, functional connectivity of the bilateral FO to the right posterior parietal cortex (PPC) increased under a visual versus proprioceptive task set. These findings suggest that the bilateral FO generally monitors manual action performance; and, moreover, that when visual action feedback is used to guide action, the FO may signal an increased need for control to visuomotor regions in the right PPC following errors.

## Significance Statement

The brain uses feedback from the senses to guide behavior and correct errors. During hand movements, this feedback can come from seen and felt hand positions. Here, we used brain scanning to show that brain regions in the frontal operculum responded to action errors in a hand–target matching task, when either seen or felt hand positions were task relevant. Furthermore, when the seen hand position had to be prioritized for the task, these regions increased their communication with the right posterior parietal cortex, which is known to guide hand movements based on visual cues. These results suggest a crucial role for the frontal operculum in monitoring hand actions; in particular, when vision is task relevant.

## Introduction

To effectively perform goal-directed action in the environment, the brain needs to monitor motor performance and detect errors, so that it can enable adaptive changes in behavior (Diedrichsen et al., 2005; Klein et al., 2007; Suminski et al., 2007; Ullsperger et al., 2014). During performance monitoring, the predicted outcome of one’s actions is compared with actual sensory feedback, and behavioral changes are initiated if a mismatch between both is detected (Ullsperger et al., 2014). The neurofunctional basis of performance monitoring and error correction has been illuminated by recent brain imaging and electrophysiological work. Specifically, a “salience network” comprising, among others, the dorsal anterior cingulate cortex, the bilateral insular cortex (IC) and the inferior frontal gyri, is assumed to integrate sensory input, responding to behaviorally salient stimuli—behavioral errors—with increased activation (Seeley et al., 2007; Sridharan et al., 2008; Ham et al., 2013; Uddin, 2021). Thereby, regions like, for example, the frontal operculum (FO; also anterior IC; Sridharan et al., 2008; Higo et al., 2011; Klein et al., 2013; Cieslik et al., 2015; Billeke et al., 2020) may signal a need for increased cognitive control to the executive control network, consisting (among other regions) of the lateral prefrontal cortices, the posterior parietal cortex (PPC), supplementary motor area (SMA), and the inferior parietal lobule (Ullsperger et al., 2010; Uddin, 2021). This network, in turn, may direct (e.g., attentional) resources to the relevant stimuli, driving behavioral adaptations (Sridharan et al., 2008; Menon and Uddin, 2010).

Notably, action performance and error may be conveyed via different sensory modalities; in manual action, for instance, via visual and proprioceptive cues about body position. In the context of body representation for action, visual and proprioceptive body position cues can be weighted depending on the current context (e.g., based on their relative relevance for the specific task at hand (van Beers et al., 1999; Sober and Sabes, 2005; Lebar et al., 2017; Limanowski, 2022). Recently, we have used virtual reality (VR) to examine this contextual sensory weighting during action under conflicting visual (virtual) and proprioceptive (real, unseen) body position feedback. Our fMRI and MEG studies (Limanowski et al., 2020; Limanowski and Friston, 2020) specifically shed light on the effects of adopting a visual versus proprioceptive attentional set during goal-directed manual action tasks, demonstrating that participants can prioritize either modality over the other; and we observed corresponding changes of neuronal gain in the respective sensory (visual and proprioceptive) brain regions.

Here, we capitalized on the novel task design of these studies. First, we aimed to investigate the neuronal correlates of performance monitoring in this ecologically valid VR-based goal-directed grasping task, and to compare the results to previous work using more abstract study designs. Second, as the original studies had demonstrated behavioral and neuronal effects of an experimentally controlled task set prioritizing vision versus proprioception, another goal of our analysis was to determine whether performance monitoring would be modality specific (i.e., involve different brain regions when vision versus proprioception was task relevant) or modality general. Based on the above literature, we expected task inaccuracy to be reflected by activity in the performance monitoring network and, potentially, also in frontoparietal attentional areas. Furthermore, in contrast to the original MEG results, which had suggested low oscillatory frequencies (in the beta band) as encoding an attentional task set (Limanowski et al., 2020), we expected high frequencies to reflect error processing (in line with assumptions of the predictive coding framework; Friston et al., 2015). We therefore reanalyzed the behavioral, fMRI, and MEG data from our above studies, correlating participants’ task performance with hemodynamic and oscillatory activity, and testing for differences in brain connectivity.

## Materials and Methods

### Participants

For this study, we reanalyzed fMRI and MEG data acquired by Limanowski et al. (2020) and Limanowski and Friston (2020). Healthy, right-handed volunteers with normal or corrected-to-normal vision participated in both experiments after providing written informed consent. The fMRI study included 16 subjects (8 females; mean age, 27 years; age range, 21–37 years), and the MEG study included 18 subjects (9 females; mean age, 29 years; age range, 21–39 years). Both experiments were approved by the local research ethics committee (University College London) and conducted in accordance with these approvals.

### Experimental design and task

Participants wore an MR-compatible data glove (1 sensor per finger; 8 bit flexure resolution per sensor; sampling rate, 60 Hz; communication with the PC via USB; Data Glove MRI, 5DT) on their right hand. The glove measured the flexion of each finger via sewn-in optical fiber cables and was carefully calibrated before scanning to fit each participant’s movement range. Recorded hand movement data were used to control a photorealistic virtual hand (VH) model, moving in accordance with the participant’s hand movements and presented as part of a virtual reality task environment. This virtual environment, consisting of the VH, a fixation dot, and task instructions, was created in the open-source 3D computer graphics software Blender (http://blender.org). The environment was presented via a projector on a screen (for details, see Limanowski and Friston, 2020; Limanowski et al., 2020).

**Figure 1.**
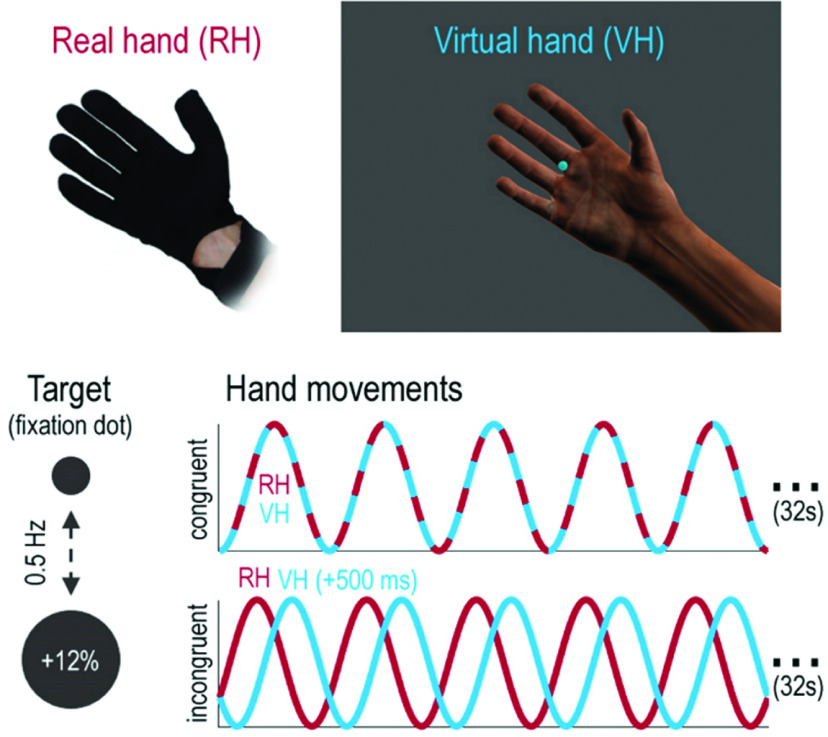
Phase-matching task. Participants controlled a photorealistic VH model with a data glove worn on their right hand. In all experimental conditions, the RH was occluded from view, while the VH was visible at all times. Participants had to match the oscillatory phase of a virtual target (fixation dot, changing its size sinusoidally at 0.5 Hz) with grasping movements (i.e., open at maximum target size, closed at minimum size). Thereby, participants were instructed to match the oscillatory phase of the target with the grasping movements of either the VH or the unseen RH (while ignoring the movements of the VH). These instructions induced a specific task set, in which either visual or proprioceptive hand movement information was task relevant. In half of all trials, RH and VH moved congruently (“congruent”), while in the other half of the trials (“incongruent”) the movements of the VH were delayed with respect to the actually executed movements (RH); this introduced visuoproprioceptive incongruence. Reprinted from Limanowski et al. (2020). Copyright Elsevier (2020) under the terms of the Creative Commons CC-BY license.

Participants were instructed to perform repetitive right-hand grasping movements, paced by oscillatory (0.5 Hz) size changes (12%) of the central fixation dot. Thus, participants had to match the fully open hand position with the biggest dot size and, conversely, the fully closed hand with minimal dot size. Choosing the fixation dot as a target for the phase matching was done to ensure fixation (for the corresponding eye tracking analyses that support this, see Limanowski et al., 2020; Limanowski and Friston, 2020) and to prevent participants from looking away from the VH—this was necessary since we wanted the visual action feedback (from the hand) to be comparable across VH and real hand (RH) conditions (while it was effectively a distractor in the RH condition). However, note that what was tracked was an oscillatory size change (i.e., a rather abstract quantity). In other words, the task was a phase-matching task (Fig. 1) rather than a visuospatial pursuit task; with this, we aimed to minimize the visual bias implicit in the design. Participants performed the task in movement blocks of 32 s (16 close-and-open movements; the last movement was signaled by brief blinking of the fixation dot), separated by 16 s rest periods during which only the fixation dot was visible. All participants trained extensively before scanning. Note that this task was not designed to investigate visuomotor adaptation or learning, but to maintain hand–target phase matching during a sustained visual versus proprioceptive attentional task set.

In half of the conditions, a lag was introduced to the movements of the virtual hand to evoke visuomotor incongruence (i.e., the virtual hand movements lagged behind the actually executed movements). In the fMRI experiment, a lag of 267 ms was introduced in the second half of each movement block; this delay length was chosen based on the results of a previous study that showed this amount of lag was well perceptible and could be adapted to with recurrent grasping movements (Limanowski et al., 2017). Since a pretest for the subsequent MEG study showed that the behavioral effects observed in the fMRI study were slightly stronger when introducing longer delays, in this experiment a lag of 500 ms was presented in separate delay blocks. However, differences in the amount of lag did not change the nature of the task; and the behavioral results were comparable across fMRI and MEG experiments (Limanowski and Friston, 2020; Limanowski et al., 2020). Before the start of each movement block, participants were instructed to match the phase of the fixation dot with either the seen virtual hand model or their unseen real hand. The virtual hand was visible in both conditions. Under incongruence, only one modality could be aligned with the target phase, which resulted in a misalignment of the other modality; when trying to align their unseen real hand with the phase of the dot, participants therefore had to ignore the movements of the virtual hand. Note that it was visual hand movement that was therefore task relevant or irrelevant, not visual information per se (which included the visually presented target dot). The task instruction (“VIRTUAL” or “REAL”) was presented 2.5 s before the start of each movement block for 2 s, and through the block, the color of the fixation dot reminded participants of the current condition. In the fMRI experiment (Limanowski and Friston, 2020), we additionally varied the visibility (high or low) of the virtual hand during half of the movement blocks. However, we found no differences in performance between different visibility levels; in our present reanalysis, there were no significant differences between visibility levels either. Therefore, we present the differential fMRI task contrasts in terms of VH versus RH task, summing over high- and low-visibility levels in each condition. In sum, despite minor technical differences between fMRI and MEG experiments, both can be described as a balanced 2 × 2 factorial design with the factors “task” (VH vs RH) and “congruence” (congruent vs incongruent).

### Behavioral data analysis

In our previous analyses, we examined the neuronal correlates of the instructed task set, and analyzed only condition-specific differences in average performance (Limanowski et al., 2020; Limanowski and Friston, 2020). In the present study, we examined the neuronal correlates of phase-matching accuracy (i.e., fluctuations around those average performances). All analyses were performed using MATLAB (MathWorks).

To quantify hand–target phase-matching accuracy/inaccuracy, we calculated the root mean square error (RMSE) of the difference between the target position (i.e., the position within the oscillatory cycle) and the position of the task-relevant hand (i.e., the position within the grasping cycle, averaging the recorded finger position data per hand). Thus, the virtual hand position was evaluated for the VH condition movements, and the real hand position for the RH condition. For construction of the fMRI/MEG regressors, we binned the resulting RMSE values into 1 s time windows, each centered on a time point of minimum or maximum target size, corresponding to the hand fully closed or opened if moved synchronously with the target. To focus on within-subject fluctuations in performance, rather than between-subject differences, the overall RMSE across the entire experiment was normalized for each single subject (i.e., minimum and maximum performance error value was equal across participants; 0 and 1, respectively). The resulting RMSE values were assigned to one regressor per experimental condition (VH congruent, VH incongruent, RH congruent, RH incongruent), and demeaned separately to reflect only variation around the condition mean. To evaluate whether phase matching differed between conditions, we calculated a two-way repeated-measures ANOVA with the factors “task set” (VH or RH) and “congruence” on the RMSE values.

The amplitude of the hand movement at each time point was calculated via a cubic spline interpolation of the respective minimum and maximum hand position values in each time window. The resulting time series was also demeaned per condition and was used as a noise regressor for the following fMRI and MEG analysis (see below). We also calculated the mean values of hand movement velocity, acceleration, and jerk per subject and condition. For each of these movement parameters (including amplitude), we calculated a two-way repeated-measures ANOVA with the factors task set (VH or RH) and congruence to evaluate condition-specific differences. Note, however, that the calculated RMSE values implicitly captured all potential differences in movement characteristics as they imply a deviation from the optimal movement trajectory; therefore, these differences are considered negligible and are reported here: Movement amplitude was found to be significantly higher for condition RH than VH in both datasets (ANOVA, main effect of task set: fMRI: *F*_(1,15)_ = 7.705, *p* = 0.014; MEG: *F*_(1,17)_ = 8.038, *p* = 0.011); in the fMRI data, movement amplitude was also significantly higher for incongruent than congruent trials (*F*_(1,15)_ = 6.499, *p* = 0.022). Mean amplitude values with associated SD for the fMRI dataset were as follows: VH_congruent = 0.607 (0.088); VH_incongurent = 0.640 (0.079); RH_congruent = 0.644 (0.098); and RH_incongurent = 0.664 (0.094). Mean amplitude values with associated SD for the MEG dataset were as follows: VH_congruent = 0.696 (0.101); VH_incongurent = 0.681 (0.108); RH_congruent = 0.722 (0.089); and RH_incongurent = 0.721 (0.119). Furthermore, in the fMRI study data (but not in the MEG study data), movement velocity was, on average, significantly higher for RH than VH (*F*_(1,15)_ = 6.726, *p *=* *0.020), and higher in trials with delayed compared with congruent seen movements (*F*_(1,15)_ = 5.044, *p* = 0.040); mean velocity values with associated SDs were as follows: VH_congruent = 0.0106 (0.0014); VH_incongurent = 0.0111 (0.0013); RH_congruent = 0.0112 (0.015); and RH_incongurent = 0.0114 (0.0015). It should be noted that, although significant, these differences were very small; there were no significant effects of acceleration or jerk. For completeness, we also tested for correlations between performance error and movement amplitude, velocity, acceleration, and jerk by calculating the Pearson correlation coefficients for each participant; and testing it for significance (i.e., significant difference from zero) with a *t* test on the group level. Similarly, we calculated the correlation between performance error and fMRI head movements (realignment parameters), via subject-level Pearson correlation and group-level *t* test, adjusted for multiple comparisons for the six realignment parameters. On average, phase-matching accuracy correlated significantly, but only weakly, with movement amplitude (for fMRI data: mean Pearson’s *r *=* *0.27; for MEG data: mean *r *=* *0.18; both *t* values_(17)_ > 5, *p* values* *<* *0.05). It also correlated significantly—again, very weakly—with velocity, acceleration, and jerk (highest *r* = 0.178, lowest *r* = 0.073; for fMRI and MEG data, all *p* values* *<* *0.05). Phase matching did not significantly correlate with the fMRI realignment parameters (all *r* values < 0.02, n.s.).

### FMRI data preprocessing and analysis

All analyses were performed using MATLAB (MathWorks) and SPM12.6 (Wellcome Trust Centre for Neuroimaging, University College London; https://www.fil.ion.ucl.ac.uk/spm/).

We reused the preprocessed fMRI data by Limanowski and Friston (2020). The fMRI data had been acquired using a 3 T scanner (Magnetom TIM Trio, Siemens), equipped with a 64-channel head coil. T_2_*-weighted images were acquired using gradient echo-planar imaging sequences (voxel size = 3 × 3 × 3 mm^3^; matrix size = 64 × 72; TR = 3.36 s; TE = 30 ms; flip angle = 90°).

We fitted a general linear model (GLM; 128 s high-pass filter) to each participant. Each condition (VH, RH) was modeled with a boxcar function as a 32 s movement block; we added a parametric modulator (1/−1) to each condition encoding the first half of each block as congruent (−1) and the second half as incongruent (1) movement periods. Additionally, we included a regressor encoding the (demeaned and convolved) RMSE values for each condition; the values were resampled to match the 3.36 s scan length before this. Regressors modeling the task instructions and movement amplitude were added to the GLM alongside the realignment parameters as regressors of no interest.

For each subject, we calculated contrast images of each RMSE regressor against the baseline. These were then entered into a group-level flexible factorial design, with the factors task (VH, RH) and congruence (congruent, incongruent), and an additional factor modeling the subject constants. To assess potential differences between congruent and incongruent movement periods, we calculated separate first-level GLMs, in which the RMSE values of the second movement half were inverted; this effectively encoded the contrast congruent–incongruent. The resulting contrast images were entered into an analogous group-level GLM, as described above.

Group-level results were assessed for statistical significance using a voxelwise threshold of *p *<* *0.05, familywise error (*p*_FWE_) corrected for multiple comparisons. We projected the resulting statistical maps onto the mean normalized structural image or rendered it on the brain template of SPM12. The unthresholded T-maps corresponding to the contrasts reported here can be inspected online at https://identifiers.org/neurovault.collection:11357. For anatomic reference, we used the SPM Anatomy toolbox (Eickhoff et al., 2005).

### MEG data preprocessing and analysis

MEG signals had been acquired using a 275-channel whole-head setup with third-order gradiometers (CTF Omega, CTF MEG International Services) at a sampling rate of 600 Hz. Following the original analysis by Limanowski et al. (2020), the MEG data were high-pass filtered (1 Hz), downsampled to 300 Hz, and epoched into trials of 2 s each (each corresponding to a full target oscillation/grasping cycle).

In the main (sensor space) MEG data analysis, we looked for spectral power differences under “steady-state” assumptions (i.e., treating the spectral profile as a “snapshot” of performance-dependent responses as manifest in quasi-stationary power spectra; Moran et al., 2008; Donner and Siegel, 2011; Friston et al., 2019). We computed trial-by-trial power spectra in the 0–98 Hz range using a multitaper spectral decomposition (Thomson, 1982) with a spectral resolution of ±2 Hz. The spectra were log transformed, converted to volumetric scalp × frequency images—one image per trial—with two spatial and one frequency dimension (Kilner and Friston, 2010), and smoothed with a Gaussian kernel with full-width at half-maximum of 8 × 8 × 4 Hz. The resulting images were entered into a GLM using a within-subject ANOVA with the respective RMSE values as a covariate (first-level analysis). As in the fMRI analysis, movement amplitude was moreover included as a covariate of no interest to capture movement-related fluctuations. Contrast images were then calculated for the accuracy covariate of each condition. These contrast images were then entered into a group-level GLM using a flexible factorial design including the two within-subject experimental factors (task and congruence), and a factor modeling the between-subject variance. The statistical parametric maps obtained from the respective group-level contrasts were evaluated for significant effects using a threshold of *p *<* *0.05, familywise error (*p*_FWE_) corrected for multiple comparisons at the peak (voxel) level.

As a *post hoc* analysis, source localization of trial-by-trial correlation of gamma-band power with performance error was performed using a variational Bayesian approach with multiple sparse priors (Litvak and Friston, 2008). Source localization was performed in the 34–88 Hz range (which was the range of effects in the spectral analysis thresholded at *p *<* *0.001, uncorrected). As we had already performed an analogous localization on the fMRI data (see above), we could use the superior spatial acuity of fMRI to improve MEG source localization [i.e., the fMRI activations (thresholded at *p *<* *0.001, uncorrected) were used as empirical (spatial) priors for the Bayesian inversion routine; Henson et al., 2010; López et al., 2014]. For comparison, we also reconstructed the sources using a Bayesian beamforming approach (Belardinelli et al., 2012). This produced very similar results [i.e., the strongest effects were localized to the bilateral inferior frontal gyri, including the FO (a further, weaker source was localized to the primary visual cortex)]. The results of this source localization were summarized as 3D images and were entered into a group-level *t* test. Since the significance of the effects on spectral responses had already been established with the sensor space analysis, the ensuing statistical parametric maps were displayed at a threshold of *p *<* *0.05, uncorrected, rendered on the smoothed average brain template of SPM. The unthresholded T-map corresponding to the source localization can be inspected online at https://identifiers.org/neurovault.collection:11357.

### fMRI functional connectivity analysis

In our main analysis (see above), we identified brain areas that showed a significant response to phase-matching inaccuracy. The fMRI and MEG results consistently highlighted the bilateral FO [while further fMRI activations were found in the dorsal premotor cortex (PMd) and the dorsolateral prefrontal cortex (dlPFC)].

In our original analyses (Limanowski et al., 2020; Limanowski and Friston, 2020), the FO did not show any task-specific effects (i.e., activity differences between VH and RH tasks) per se, and neither did the SMA or the dlPFC. However, following the clear response of the FO to poor task performance in general, we now asked whether these areas would change their connectivity to other (potentially, task relevant) brain areas depending on whether the inaccuracy was registered during the VH or RH task [i.e., while participants focused either on visual (VH) or proprioceptive (RH) action feedback].

To answer this question, we used psychophysiological interaction (PPI) analysis for fMRI data. This analysis aims to explain coupled neuronal activity among brain areas in terms of an interaction between psychological factors (the specific task condition) and physiological factors (the BOLD signal time course in the region of interest; Friston et al., 1997; O’Reilly et al., 2012). The resulting PPI reveals voxels in the brain that increase their connectivity with a specific seed region in a given context (e.g., in a specific task condition). Note that task-dependent changes in connectivity per se (i.e., between VH and RH task sets) were identified in both fMRI and MEG datasets (Limanowski et al., 2020; Limanowski and Friston, 2020). However, in the fMRI data, the SPM approach allowed us to select a spatially isolated volume of interest (i.e., voxels from the FO) that was not part of the original connectivity analysis. This was not analogously possible for the MEG data, which in the original connectivity analysis were already modeled on the whole-scalp level (Limanowski et al., 2020). Therefore, we limited the connectivity analysis to the fMRI data.

For the PPI analysis, we calculated separate GLMs with concatenated runs for each participant, and thus identified subject-specific peaks of the main effects observed on the group level. The individual peaks were defined as the maximum effect within a 10-mm-radius sphere of the respective group-level maximum (Table 1). From these individual peaks, we extracted the BOLD signal of the seed regions as the first eigenvariate of activity across all voxels in a 4-mm-radius sphere centered on the participant-specific peak. For three subjects where no effect could be identified for the specific PMd seed region as well as one case where no effect was found for the dlPFC region, we resorted to the group-level maximum for seed region localization.

**Table 1:** Significant (*p*_FWE_ < 0.05) activations for all reported fMRI contrasts

Anatomical location	Voxels	MNI			Peak *T*	Peak *p*_FWE_
		*x*	*y*	*z*		
Correlation with phase-matching inaccuracy						
L. Insula (FO)	1	−26	20	12	5.92	0.012
L. Superior frontal gyrus (PMd)	13	−16	4	70	5.90	0.014
L. Middle frontal gyrus/frontal pole (dlPFC)	2	−26	44	24	5.80	0.018
R. Insula (FO)	3	30	22	12	5.72	0.023
Correlation with phase-matching accuracy						
L. Precentral and postcentral gyrus (M1)	6	−32	−18	38	6.18	0.006
	3	−28	−22	66	5.78	0.019
	1	−24	−24	62	5.49	0.044

L, Left; R, right.

**Figure 2.**
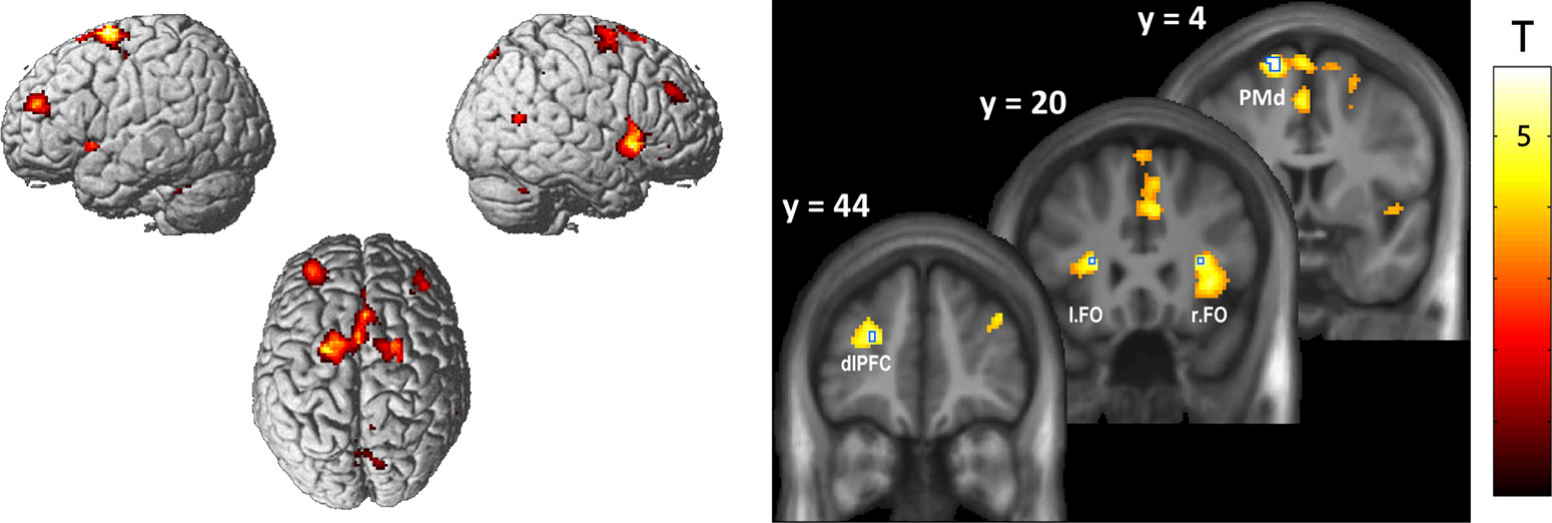
BOLD signal increases related to phase-matching inaccuracy. The renders (left) and slice overlays (right) show brain areas in which hemodynamic activity was correlated with the relative inaccuracy of hand–target phase matching (displayed at *p* <* *0.001, uncorrected). Significant activations (*p*_FWE_ < 0.05; voxels outlined in blue on the slice overlays) were located in the bilateral FO, the left SMA, and the left dlPFC.

The SPM12.6 PPI routine was then used to form the interaction between the psychological factor and the summarized BOLD signal time course of the seed region. Note that while the seed regions were identified per their significant response to phase-matching inaccuracy (Fig. 2), our psychological factor was the task set (i.e., the instructed hand modality at the beginning of each movement block; VH vs RH, pooled over different levels of virtual hand visibility; see above). After forming the interaction term, a second GLM was constructed for each participant, including the interaction, the extracted signal of the seed region, the task set, and the realignment parameters as regressors of no interest.

On the group-level, the connectivity of the bilateral FO was evaluated using a paired *t* test (i.e., a GLM including the PPI contrast images of the left and right FO of each participant, and another factor modeling the between-participant variance). We also tested whether the other two regions showing significant responses to phase-matching inaccuracy (PMd and dlPFC) would exhibit connectivity changes, using a similar approach.

## Results

### Behavioral results

In both studies, participants reported allocating their attention to the respective instructed hand, which is in line with the assumption that the task instructions would create a visual versus proprioceptive task set. Both VH and RH tasks were perceived as comparably difficult, with the incongruent conditions being judged as more difficult in each case. Moreover, in both studies, participants were able to follow the task instructions [i.e., to keep the grasping movements of the instructed modality (vision or proprioception) significantly better aligned with the phase of the dot than the noninstructed modality]. See the original studies (Limanowski et al., 2020; Limanowski and Friston, 2020) for details.

Our analysis was aimed at identifying the neuronal correlates of fluctuations of performance (i.e., fluctuations around condition-specific mean performances). For completeness, we still report the condition-specific RMSE means and SDs, as follows: for the fMRI dataset: VH_congruent = 0.265 (0.045); VH_incongruent = 0.244 (0.041); RH_congruent = 0.273 (0.058); and RH_incongruent = 0.257 (0.061); for the MEG dataset: VH_congruent = 0.213 (0.045); VH_incongruent = 0.244 (0.040); RH_congruent = 0.206 (0.041); and RH_incongruent = 0.322 (0.095). Overall, participants performed slightly better in the VH than the RH task; although this difference was statistically significant only for the MEG study data (ANOVA; main effect of task set: *F*_(1,17)_ = 7.136, *p* = 0.016). In the MEG study, RMSE was significantly higher for incongruent than congruent trials in both datasets (ANOVA: *F*_(1,17)_ = 30.026, *p* < 0.001); in the fMRI data, RMSE was higher for congruent than incongruent trials (*F*_(1,15)_ = 6.820, *p* < 0.05). In sum, the behavioral results and self-reports showed that participants could follow the task instructions, that this induced the desired cognitive-attentional task set, and that behavior in the VH versus RH tasks was comparable across fMRI and MEG studies.

### FMRI results

In our main fMRI analysis, we sought to identify brain regions in which neuronal activity correlated with phase-matching accuracy/inaccuracy (Fig. 2). A significant (*p*_FWE_ < 0.05) main effect of inaccuracy was observed in the bilateral FO, the left dorsal premotor cortex (PMd; at uncorrected thresholds, this activation cluster spanned to the left SMA), and the left dlPFC (Table 1, Fig. 2). More liberal thresholds (*p *<* *0.001, uncorrected) revealed further activation clusters in the right middle and superior frontal gyri, the precuneus, the midcingulate cortex (MCC), the right middle temporal gyrus (MTG), and bilaterally in the cerebellum (Fig. 2, compare the render). Conversely, a significant main effect of accuracy was found in the left pre- and postcentral gyrus, corresponding to the primary motor cortex (M1). No other comparisons (i.e., contrasting the effects of accuracy among task conditions, delay, or visual salience levels; see Materials and Methods) yielded significant effects. At uncorrected thresholds (*p *<* *0.001), voxels in several brain areas showed a stronger correlation with task inaccuracy under the VH task than under the RH task; namely, in the MCC, the bilateral FO, the right MTG, the left cerebellum, the right dlPFC, and the bilateral PPC (peak within the intraparietal sulcus).

### MEG results

**Figure 3.**
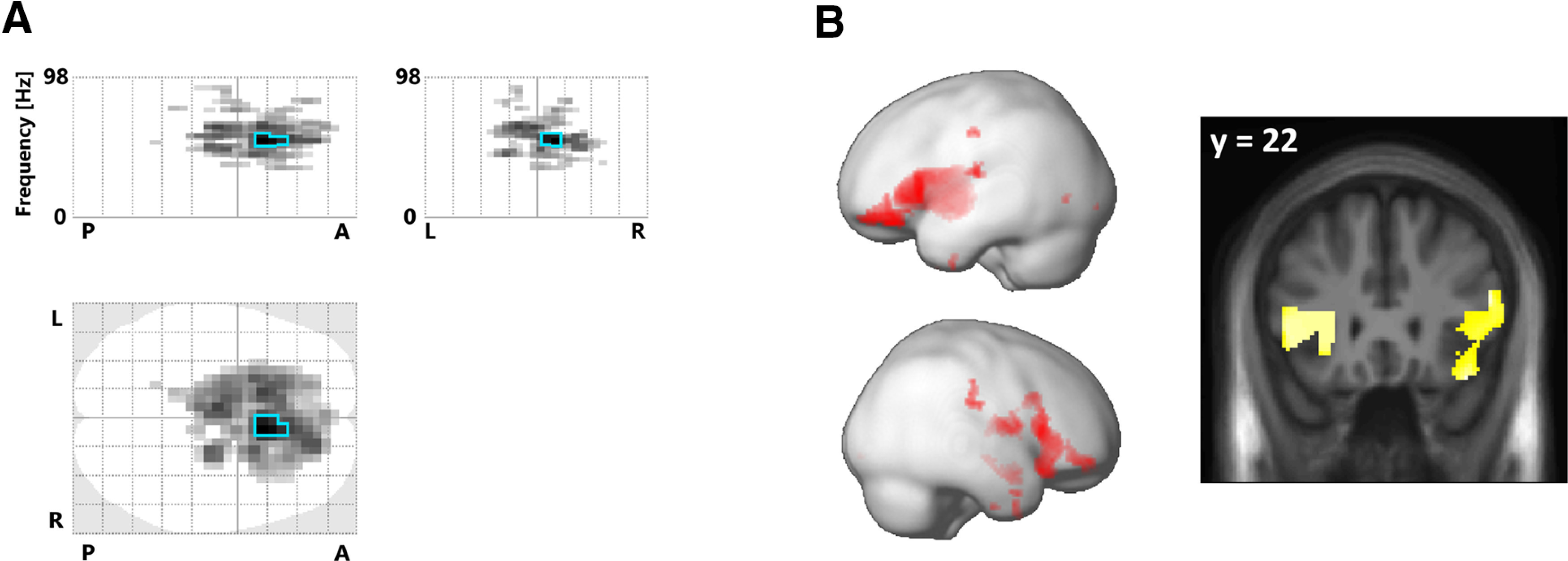
Spectral gamma power increases related to phase-matching inaccuracy. ***A***, The “glass brain” (maximum intensity) projections show the sensor level scalp frequency maps of spectral power correlated with the relative inaccuracy of hand–target phase matching (the darkest voxels show the strongest effect along the respective projection; the maps are thresholded at *p *<* *0.001, and effects significant at *p*_FWE_ < 0.05 are outlined in blue; the top plots have one frequency dimension, 0–98 Hz, and one spatial dimension. P-A, Posterior-anterior; L-R. left-right. The bottom plot has two spatial dimensions. ***B***, Renders (left) and slice overlays (right) showing the corresponding source localization of the spectral correlation to regions around the FO.

The MEG sensor space analysis revealed that phase-matching inaccuracy was associated with significantly increased spectral power in the gamma frequency range over midfrontal sensors (main effect; peak at 52 Hz, *T* = 5.31, *p*_FWE_ < 0.05; Fig. 3*A*). These spectral effects were source localized to the bilateral inferior frontal gyri, including the bilateral FO (Fig. 3*B*). No other spectral power comparisons yielded statistically significant results; but there was a statistical trend suggesting inaccuracy was associated with reduced alpha (8 Hz) power over posterior sensors (*T* = 4.61, *p*_FWE_ = 0.069).

### Functional connectivity analysis

The above fMRI activations and source-localized MEG gamma power consistently suggested that periods of poor phase matching activated the bilateral FO, in line with previous literature that had established the role of this region in error processing and performance monitoring (see Introduction). However, we did not find any significant difference between conditions (i.e., between visual and proprioceptive task sets). Therefore, we next performed a connectivity (PPI) analysis on the fMRI data to explore whether task-relevant brain areas would change their connectivity to the FO depending on the instructed task condition (VH or RH).

**Figure 4.**
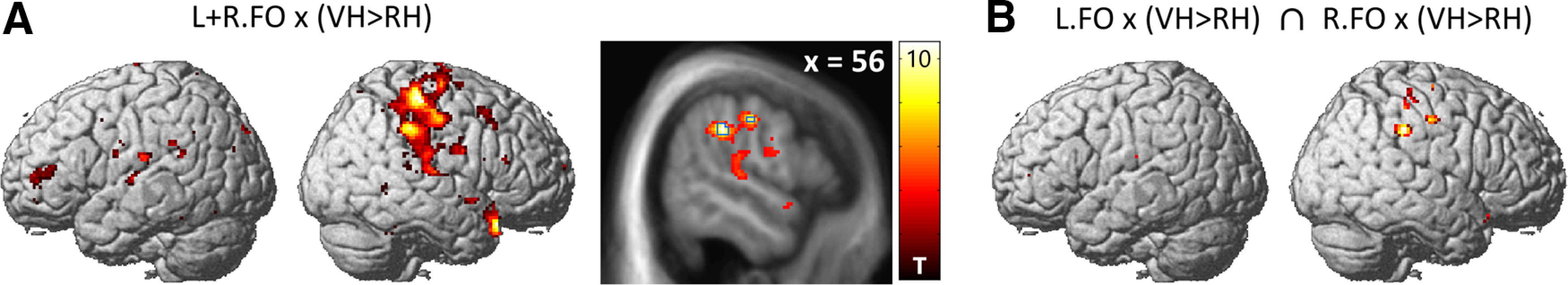
Task-dependent connectivity changes of the bilateral FO. ***A***, Brain areas showing increased coupling with the bilateral FO during the VH task relative to the RH task (displayed at *p *<* *0.001, uncorrected). The strongest effects were located in the right IPL (voxels significant at *p*_FWE_ < 0.05 are outlined in blue). ***B***, A corresponding null conjunction contrasts confirmed this increased task-dependent coupling with the right IPL for the left and right FO independently (each PPI contrast thresholded at *p *<* *0.001, uncorrected).

This analysis revealed a significantly increased coupling of several brain areas with the bilateral FO during the VH task > RH task, most strongly expressed in the right inferior parietal lobe (IPL; Fig. 4*A*, Table 2). The increase in coupling with the right IPL was evident for both the left and right FO independently, as revealed by an additional “null” conjunction analysis (Fig. 4*B*; a conjunction of voxels activated in the PPI with the left FO and PPI with the right FO, each thresholded at *p *<* *0.001, uncorrected). Correspondingly, there were no significant differences in coupling between the left and right FO. A supplementary analysis testing for potential coupling differences with the FO during VH_incongruent versus VH_congruent yielded no significant effects either. There were no significant connectivity changes with the FO under the RH task > VH task. No significant changes in connectivity were observed in analogous analyses calculated for the PMd or the dlPFC, with the other two brain regions showing significant effects in the main analysis (see above).

**Table 2:** Brain areas showing significant (*p*_FWE_ < 0.05) coupling increases with the bilateral FO during the VH task > RH task

Anatomical location	Voxels	MNI			Peak *T*	Peak *p*_FWE_
		*x*	*y*	*z*		
R. IPL/SMG	15	56	−32	38	10.71	0.002
R. Postcentral gyrus (S1)	4	54	−14	44	9.26	0.012
R. Precentral gyrus (M1)	1	4	−24	48	9.02	0.017
R. Temporal pole	2	52	20	−22	8.83	0.022
	2	40	22	−24	8.68	0.027

R, right.

## Discussion

We used data from a virtual reality-based hand–target phase-matching task to identify the hemodynamic and oscillatory correlates of performance (i.e., phase-matching accuracy) monitoring under instructed task relevance of visual or proprioceptive hand position feedback. The specific design of this task, with continuous goal-directed movements and the experimentally controlled switching of attentional task set, created a novel, ecologically valid context for performance monitoring.

Our main result was a general, modality-independent response of the bilateral FO to poor phase-matching accuracy, as evident from the increased BOLD signal levels and increased source-localized gamma power. Furthermore, connectivity of the bilateral FO to the right PPC/IPL increased while participants executed the phase-matching task with the visible virtual hand, compared with when they executed it with the real, unseen hand.

The observed general BOLD signal increase in the bilateral FO with task (phase-matching) inaccuracy replicates observations of previous studies using more abstract study designs, where the BOLD signal in the FO increased in response to performance errors. For instance, the FO was activated by error trials versus correct trials in the Simon task (Danielmeier et al., 2011; Ham et al., 2013), in an antisaccade task (Klein et al., 2007), and in a flanker task (Eichele et al., 2008). Similarly, FO error-related BOLD signal increases were observed in visuomotor adaptation tasks (Grafton et al., 2008) and in response to tactile “oddball” stimuli (Allen et al., 2016). Some studies found activation of the FO correlated positively with task performance (Bunge et al., 2002; Wager et al., 2005). This could, however, be explained with a general underlying function of FO activation in performance monitoring; acting not as an error signal per se, but as part of a mechanism to improve performance in response to errors (Eichele et al., 2008). Thus, it has been proposed that neuronal activity in the FO may indicate the need for increased allocation of attentional resources to specific stimuli to achieve task-appropriate behavior (Ham et al., 2013; Langner and Eickhoff, 2013; Cieslik et al., 2015; Uddin, 2021). Additionally, because of the reciprocal connections of the FO to multiple sensory, limbic, and association areas (Sridharan et al., 2008), it may act as crucial “relay” station for switching between different task-relevant networks (e.g., switching from default network to executive control network; Klein et al., 2007; Sridharan et al., 2008; Menon and Uddin, 2010; Ullsperger et al., 2010).

The spectral correlates of task inaccuracy were expressed in the gamma frequency range, thus confirming the potential role of high-frequency oscillations for conveying error signals (Friston et al., 2015). Furthermore, notably in agreement with the BOLD signal increases, they were source localized to the bilateral FO (as part of larger sources in the inferior frontal gyrus). A general correspondence and spatial colocalization of the BOLD signal and gamma power has been established in previous studies (Brovelli et al., 2005; Foucher et al., 2003; notably, including colocalization of responses in the insula, Castelhano et al., 2014). Our findings also align with previous studies that reported increases in intracranially recorded gamma activity in the FO following (stop-signal) task errors (Bastin et al., 2017). Moreover, gamma-band activity per se is often interpreted as indicating enhanced processing of attended (e.g., task-relevant) sensory information (Jensen et al., 2007; Hipp et al., 2012; Siegel et al., 2012; Clayton et al., 2015). In other studies, increased gamma power (over mid-frontal sensors) during response competition has been interpreted as indicating increased cognitive control (Grent-‘t-Jong et al., 2013). A Granger causality analysis by Chand and Dhamala (2017) suggested that, during perceptual decision-making, the FO may exert causal influence over frontoparietal areas within the gamma band.

In sum, and in light of the above literature, our fMRI and MEG results suggest that the FO is involved in performance monitoring during goal-directed hand movements, corroborating its role as suggested by previous studies in other, nonmotor tasks. Notably, while most of the above studies used trial-by-trial designs, our study featured continuous movements; thus, our results complement previous literature in showing that the FO shows similar responses in task settings requiring “online” performance monitoring and adjustment during manual actions. Specifically, we propose that FO activation (expressed through BOLD signal and gamma power increase) may have indicated a reaction to task inaccuracy or error, and a corresponding need for behavioral adjustment. Tentatively, this interpretation is supported by the fact that posterior alpha power behaved opposite to gamma (i.e., it decreased with increasing inaccuracy, although this effect did not reach statistical significance; see Results). It is well established that posterior alpha inversely correlates with attention and task engagement (Yamagishi et al., 2003; Sauseng et al., 2005; Thut, 2006; Bacigalupo and Luck, 2019).

In addition to increased activation of the bilateral FO, our fMRI connectivity analysis revealed that these areas also increased their functional coupling with the right PPC (peak located in the IPL) during the VH task (phase matching with vision) compared with the RH task (phase matching with proprioception). An fMRI study by Higo et al. (2011) used a task requiring attention to faces, houses, or body parts, and found that the FO increased its functional coupling with visual areas processing the respective task-relevant stimulus category. In our case, the connectivity increase of the FO was not with primary and secondary visual cortices, which had shown task-dependent (attentional set) activity increases in our previous studies (Limanowski et al., 2020; Limanowski and Friston, 2020). Instead, FO coupling increased with the IPL of the right PPC; an area that is involved in more high-level processes including multisensory and sensorimotor integration, and visuospatial attention (Wolpert et al., 1998; Andersen and Buneo, 2002; Malhotra et al., 2009).

We propose that this result is related to the fact that visual hand movements were task relevant in the VH task, but had to be ignored in the RH task (where phase matching was done with proprioception). Thus, visual hand movements were essential for correcting phase-matching error in the VH task, but irrelevant in the RH task. Notably, FO connectivity was not significantly different during periods of visuoproprioceptive incongruence; neither did we find significant differences between congruent and incongruent conditions in the main fMRI GLM analysis. This suggests that the observed VH > RH task-dependent connectivity difference was related to the task-relevant hand feedback modality being vision > proprioception per se, rather than to congruence/incongruence between vision and proprioception. This interpretation fits with previous work showing that the right PPC, specifically areas in the right IPL, are critical hubs for executing and correcting visually guided arm movements (Desmurget et al., 1999; Culham et al., 2003; Wenderoth et al., 2004; Culham and Valyear, 2006; Ogawa et al., 2007; Lane et al., 2011).

Potentially, this effect might have been enhanced by intrinsic differences between the modalities in relation to visuoproprioceptive integration and error detection [i.e., it might have been easier for participants to notice a phase-matching error when focusing on visual action feedback (VH task) than when focusing on proprioception (RH task)]. This could have been because of visual body position estimates being intrinsically less variable than proprioceptive ones (van Beers et al., 1999), and because the visual body position was easier to compare with, and integrate with, the visually presented target (in the VH task) than the proprioceptive body position (in the RH task). However, note that the target quantity was not visuospatial but abstract (i.e., the oscillatory growing-and-shrinking phase of the fixation dot). When we lowered the statistical threshold of the main GLM analysis to *p *<* *0.005, uncorrected, the bilateral FO (the PPI seed regions) and the right PPC (the PPI target region) showed a stronger correlation with task inaccuracy under the VH task compared with the RH task. Although this was a weak effect, it could mean that, overall, errors were more easily processed (in those areas) in the VH task. Interestingly, participants performed slightly worse in the RH than in the VH task, which could support the interpretation that proprioceptive performance monitoring was less efficient than when vision was used. This may also fit with previous reports of increased BOLD signal in the FO for error trials of which participants were aware, compared with unaware errors (Klein et al., 2007; Harsay et al., 2018). Specifically, Harsay et al. (2018) also observed increased functional connectivity of the FO to the PPC [bilaterally, in addition to the bilateral primary somatosensory cortex (S1)] during aware > unaware errors.

In sum, we suggest that the increased connectivity between the FO and the right PPC during the VH > RH task indicates that the FO signals an increased need for control and attentional and/or behavioral adjustment (following poor performance) to visuomotor regions in the right PPC, which could also be related to how easily those performance deficits could be detected.

The above speculations could explain why we did not observe any connectivity increases of the FO during the RH > VH task. Accordingly, this could be because participants were less aware of their phase-matching accuracy/inaccuracy when performing the task with the unseen real hand. Future work should evaluate this possibility.

In addition to activations in the bilateral FO, we also found BOLD signal increases because of poor phase matching in the PMd (spanning to the SMA at uncorrected thresholds) and in the dlPFC (at the junction of middle frontal gyrus and frontal pole). Both areas have been strongly implied in performance monitoring in other contexts, albeit implying the SMA rather than the more lateral PMd (Ullsperger et al., 2014). The SMA has been shown to respond to unexpected stimuli (e.g., surprising action outcomes; Sakai et al., 1999; Krakauer et al., 2004; Ullsperger et al., 2010; Scangos et al., 2013). Furthermore, BOLD signaling in the SMA and the PMd has previously been reported to correlate with positional error in a visuomotor learning task (Grafton et al., 2008) and in continuous hand–target tracking (Limanowski et al., 2017). In line with the interpretation provided in these studies, the PMd (and, uncorrected, SMA) activation we observed may indicate an updating of movement plans in response to poor detected phase matching. Similarly, the lateral PFC is considered a crucial part of the sensorimotor hierarchy (Benchenane et al., 2011; Sokhadze et al., 2012) and is thought to contribute to performance monitoring and error detection (e.g., by preparing attentional task sets and comparing behavioral output against them; Ullsperger and von Cramon, 2004; Danielmeier et al., 2011; Cieslik et al., 2015; Smith et al., 2019). In our experiment, the dlPFC activation could imply similar underlying “high-level” functions.

Conversely, we observed that BOLD signal in the contralateral M1 correlated positively with task accuracy. This effect could be related to the fact that higher task accuracy coincided with more pronounced hand movements and, thus, also with associated differences in movement velocity, acceleration, and jerk. Future work will have to clarify whether differences in movement trajectories contribute to differential M1 activation, as observed here. However, we had included movement amplitude as a regressor of no interest in our first-level GLMs, which should have largely accounted for this potential bias. Alternatively, this observation also aligns with the known role of M1 in motor learning (Hardwick et al., 2013; Spampinato and Celnik, 2017; Panico et al., 2021), with previous findings that M1 activity correlated with visuomotor adaptation performance (Della-Maggiore and McIntosh, 2005) or with visuomotor target-tracking performance (Ogawa et al., 2006), and with the fact that a perturbance of the M1 via transcranial magnetic stimulation resulted in reduced sensorimotor adaptation (Orban de Xivry et al., 2011).

Our results should be compared with previous studies with some caution, since our task was designed around continuous movements; therefore, we could not isolate specific time points—and neuronal correlates—that would clearly correspond to specific cognitive or motor processes like, for example, error detection or correction. Future variations of our task design should therefore try to validate our interpretation.

In conclusion, our results suggest a critical role for the bilateral FO in performance monitoring during goal-directed manual action, and that, following errors in visually guided manual action specifically, the FO may signal an increased need for control to visuomotor regions in the right PPC.

## Synthesis

Reviewing Editor: Satu Palva, Helsingin yliopisto Decisions are customarily a result of the Reviewing Editor and the peer reviewers coming together and discussing their recommendations until a consensus is reached. When revisions are invited, a fact-based synthesis statement explaining their decision and outlining what is needed to prepare a revision will be listed below. The following reviewer(s) agreed to reveal their identity: NONE. Note: If this manuscript was transferred from JNeurosci and a decision was made to accept the manuscript without peer review, a brief statement to this effect will instead be what is listed below.

I have reviewed your revised manuscript and foreseen that all concerns raised by previous reviewers were adequately addressed.
